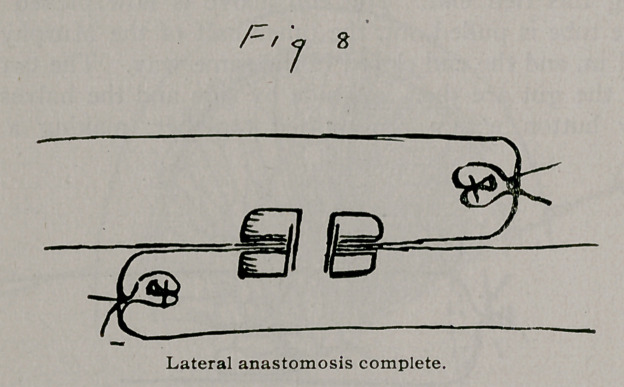# Intestinal Resection and Anastomosis; A Rapid and Safe Method1Read at the thirty-ninth annual meeting of the Medical Association of Central New York, at Syracuse, October 16, 1906.

**Published:** 1907-04

**Authors:** W. L. Wallace

**Affiliations:** Syracuse, N. Y.


### BUFFALO MEDICAL JOURNAL.

VOL. LXII. APRIL 1907. No. 9

### ORIGINAL COMMUNICATIONS.

### Intestinal Resection and Anastomosis; A Rapid and Safe Method.[1]

By W. L. WALLACE, M. D., Syracuse, N. Y.

A PATIENT who needs an intestinal resection is always in poor and often in desperate condition. Shock is profound from obstruction, strangulation, distention and absorption. An operation is therefore required which will give the quickest possible relief from these dangers and add as little shock of its own as possible. In other words, the relief from the shock of the obstruction must be more rapid than the addition of the shock of the ether, the cutting, the exposure and the hemorrhage.

Such an operation must relieve the distention and remove the poisonous fluids which are being absorbed, and make a safe resection and anastomosis without loss of blood and without soiling the peritoneum, and with great rapidity. Each surgeon develops a technic from his own observation and experience. My present method in a desperate case is about as follows:

First, I assign the general care of the patient to a reliable medical or surgical colleague whose duty is to wash out the stomach, assist the anesthetist, and attend to salines, drugs and heat; and I even find that washing out the quarts of fecal-like gases and fluids that are in the stomach at the beginning of the operation is not sufficient, but that it is necessary to keep washing them out during and at the close of the operation.

The abdomen being open, the first step is to find the obstruction. The ileocecal region is explored, and if it is collapsed the trouble is in the small intestine and the collapsed gut is then followed up to the obstruction. If the ileocecal region is distended, the obstruction is in the large intestine and the sigmoid is next investigated.

1. Read at the thirty-ninth annual meeting of the Medical Association of Central New York, at Syracuse, October 16, 1906.

To illustrate the method of resection and anastomosis, let us find an obstruction and strangulation by a band in the middle portion of the ileum, and let the mesentery be more or less damaged by thrombosis so that it will have to be removed. *Fig. 1.*

Fig. 1.

Gut distended above and collapsed below obstructing band (a).

The obstruction being found, and a resection necessary, the next step is to decide how much bowel must be removed. The gut is usually damaged above the obstruction for a greater distance than at first appears and after having removed 6 inches, I have, on more careful examination, cut away 12 inches more. In order to go well above the damaged area let us decide in this case to remove 12 inches.

We are now ready to relieve the distention and stop the absorption by emptying the gut above. Moynihan passes a glass tube into the gut above the obstruction, drawing more and more of the intestine upon the tube as it empties itself, sometimes getting 10 to 15 feet upon a tube six or eight inches long. In this way the poisonous pressure is removed leaving the intestines above the obstruction empty and contracted, making room and suitable conditions for a rapid repair and an easy closure of the abdomen. With the instruction gained by using the Moynihan method, I proceed as described in the next paragraph.

The gut is drawn out of the abdomen above the obstruction without pulling away the band or adhesion, and the amount to be resected is decided upon, which in this case is twelve inches. This portion is gently emptied into the gut above by pressure with the fingers and is then clamped across about a foot above the obstruction with two pairs of common pedicle forceps, and cut through between the clamps. The upper end is now drawn away from the wound and a tube, such as a common stomach tube or a rectal tube, is passed at least twelve inches into the end of the gut and tied in; and the gut is allowed to drain through this tube while the operation proceeds, more and more of the gut being drawn by the assistant upon the tube as it empties itself. *Fig. 2.*

Fig. 2.

Tube in gut for drainage. At the right the gut is being drawn upon tube.

The obstruction being found, the amount to be removed decided upon and the gut emptied, we are ready to resect. The piece of the gut to be removed is brought up and the band or adhesion which caused the obstruction is pulled away. The bowel is now clamped below the obstruction for cleanliness and convenience in handling and is cut across between the clamps Much time and blood will now be saved, if we look through the mesentery holding an electric light behind it, unless it is thin, in order to be able to see the exact distribution of the bloodvessels. As we look upon the mesentery without the light behind, it appears opaque, and often we cannot see the vessels until they bleed; and at times, after having made a V cut into the mesentery without the lamp, I have found that I have not gone exactly straight, but have undermined one side, damaging the bloodvessels of the adjacent healthy gut, necessitating a wider resection. With a lamp behind, it is easy to throw a ligature around the arteries at the apex of the V and to tie all vessels before cutting.

The posterior border of the mesentery attached to the back of the abdomen is about six inches long. Its anterior border at the small intestines is about twenty feet long. Therefore, each inch of its posterior border supplies about forty inches of its anterior border, so that the vessels at the apex of the V which supply the twelve inches of gut to be resected are very few, and the damage done by going half an inch to one side or the other may be very considerable; and on the other hand, when we are able to see the exact blood distribution, the control of hemorrhage at the apex of our V is easy and sure. *Fig 3.* The lamp

Fig. 3.

A thick mesentery. Lamp held behind at left showing bloodvessels by transmitted light. On right, by reflected light, bloodvessels indistinct.

behind may be covered with gauze, and this method saves much time and blood and uncertainty. The surgeon is confident. He can see that he has not cut off the blood supply of the edges of his repair. If the mesentery is very thin and transparent it is easy to hold it up and look through it without the lamp.

The damaged intestine and mesentery are removed and we are now ready for an anastomosis. *Fig. 4.* I can do this

Fig. 4.

Gut resected. Ends ready for anastomosis.

most rapidly and satisfactorily by making a lateral anastomosis with a Murphy button, applying Hartley's method. I bring up the two ends which are to be joined, take off the clamp which is on the end below and drop into the end of the gut one of the halves of a Murphy button. Then I close this end, first tying it where it was clamped and then, with a fine purse-string suture, inverting this tied end. The end above is now picked up, the drainage tube is pulled out, the other half of the Murphy button dropped in, and the end closed in the same way. The two closed ends of the gut are then laid side by side and the halves of the Murphy button within are pushed together, making a lateral

Fig. 5.

Half Murphy button dropped into gut. 1. End of gut tied. 2. Purse-string suture ready to invert tied end.

anastomosis. This is done as follows: one end of the gut is picked up and the half of the Murphy button within is found and grasped in such a way that its cylinder is made to press against the side of the gut one or two inches from the closed end. With a knife the bowel is nicked over the end of the cylinder of the button which is thus allowed to push through. The same is done with the other half of the button which is in the other closed arm of gut. The two halves of the button whose cylinders are thus protruding from within the gut, are pushed together and the anastomosis is complete. The sides of the mesenteric V are stitched together, and the abdomen is closed. *Figs. 5, 6, 7, 8.*

Fig 6.

1. Tied end inverted as (2) purse-string suture is tied. 3. Murphy button pushed against side of gut which is cut with cross to allow cylinder to protrude.

Fig. 7.

Half of Murphy button in place.

This method is very rapid. I can make this lateral anastomosis much quicker than I can do an end to end anastomosis. In addition to the time saved, I think this method much safer. In any end to end anastomosis, there is danger of the blood supply to some point of the repair being shut off. In this lateral method the possibly doubtful ends are turned nearly one inch

Fig 8

Lateral anastomosis complete.

into the gut. In doing an end to end anastomosis with a Murphy button, it is necessary to use a purse-string suture, puckering a gut one to two inches in diameter around a cylinder one-half inch in diameter, being sure that the mucous membrane does not pout at any point and that peritoneum is in exact contact with peritoneum. Moreover, the puckered mass is so uneven, with the mesentery crowded under at one side that considerable strain comes upon the button and some little point is liable to slip. At any rate, in doing an end to end anastomosis with a Murphy button, I have felt safer after reinforcing with sutures.

With the lateral anastomosis, the holes through which the cylinder ends protrude are of the exact size of the cylinders and no puckering string or stitches are needed, only two smooth and even layers of the gut being held between the parts of the button, with no undue or uneven tension on the spring and with the surfaces of gut in contact for a good distance from the edge of the communicating opening.

SUMMARY.

The stomach having been washed out and the other preparations made, the obstruction is found and the gut clamped above and below and cut across, a tube being tied in above to allow the distended bowel to empty itself. A light is held behind and the gut is resected. An anastomosis is made by dropping a half of a Murphy button into each arm and closing the ends. and then pushing the two halves of the button together.

620 East Genesee Street.

## Figures and Tables

**Fig. 1. f1:**
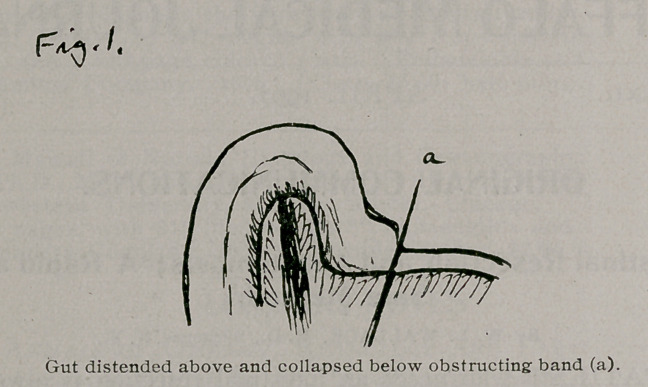


**Fig. 2. f2:**
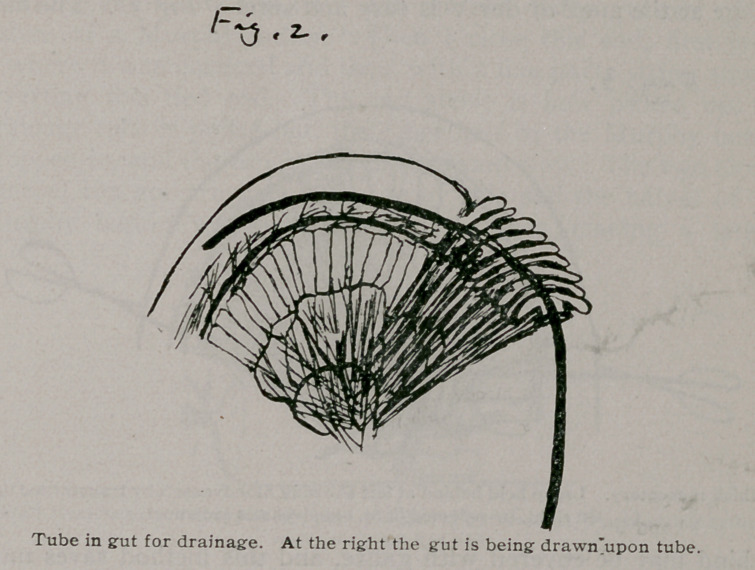


**Fig. 3. f3:**
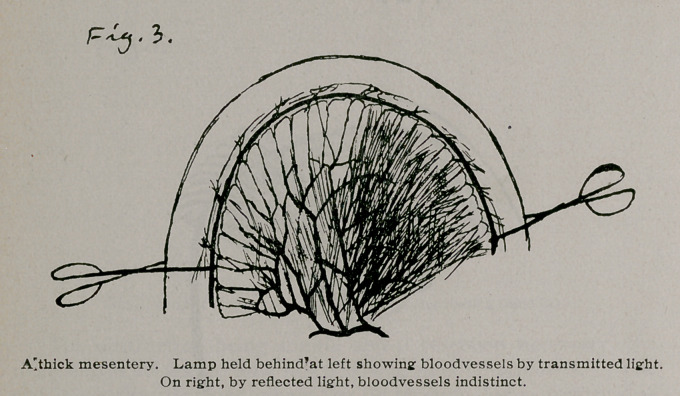


**Fig. 4. f4:**
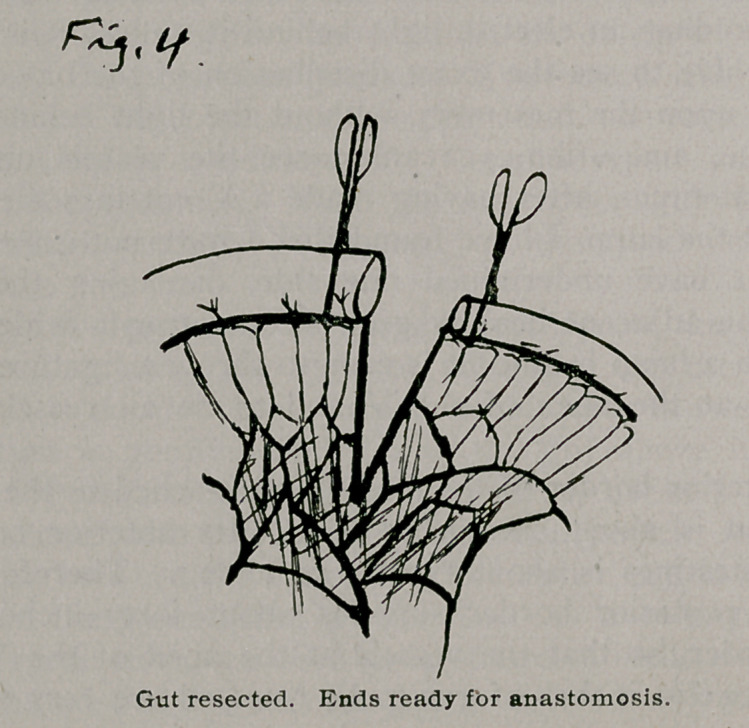


**Fig. 5. f5:**
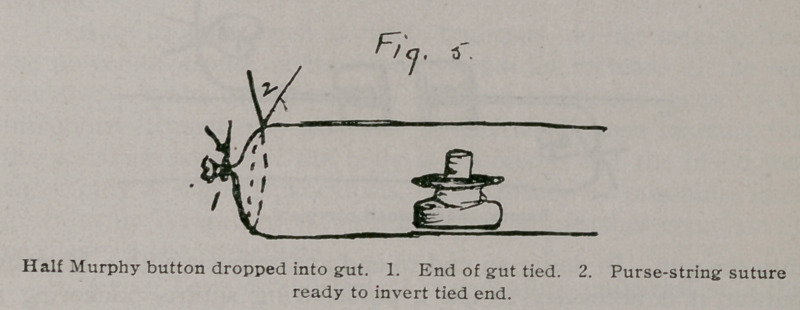


**Fig. 6. f6:**
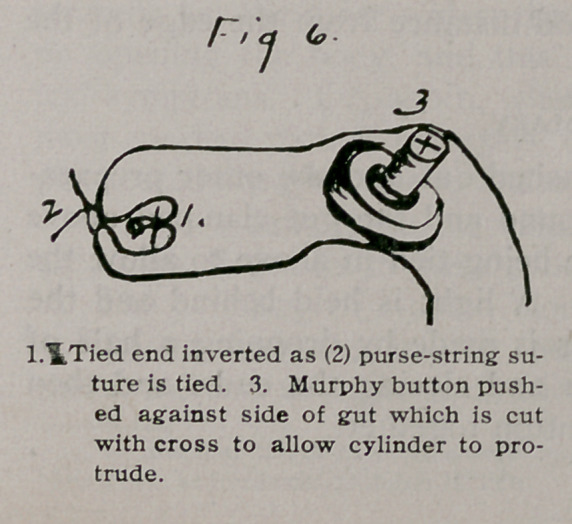


**Fig. 7. f7:**
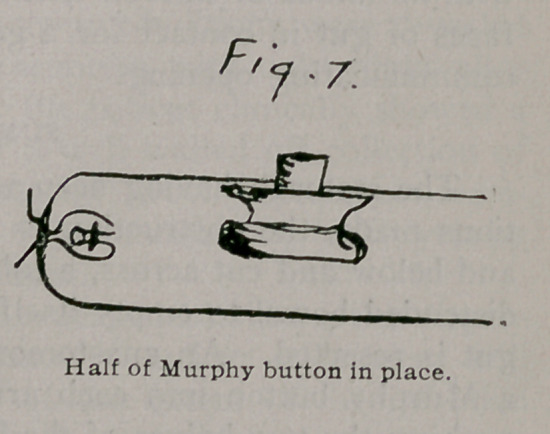


**Fig. 8. f8:**